# Electromyographic typing gesture classification dataset for neurotechnological human-machine interfaces

**DOI:** 10.1038/s41597-025-04763-w

**Published:** 2025-03-15

**Authors:** Jonathan Eby, Moshe Beutel, David Koivisto, Idan Achituve, Ethan Fetaya, José Zariffa

**Affiliations:** 1 KITE Research Institute, Toronto Rehabilitation Institute - University Health Network, Toronto, Ontario M5G 2A2 Canada; 2 Institute of Biomedical Engineering, University of Toronto, Toronto, Ontario M5S 3G9 Canada; 3 Alexander Kofkin Faculty of Engineering, Bar-Ilan University, Ramat Gan, 5290002 Israel; 4 Rehabilitation Sciences Institute, University of Toronto, Toronto, Ontario M5G 1V7 Canada; 5 Edward S. Rogers Sr. Department of Electrical and Computer Engineering, University of Toronto, Toronto, Ontario M5S 3G4 Canada

**Keywords:** Biomedical engineering, Somatic system

## Abstract

Neurotechnological interfaces have the potential to create new forms of human-machine interactions, by allowing devices to interact directly with neurological signals instead of via intermediates such as keystrokes. Surface electromyography (sEMG) has been used extensively in myoelectric control systems, which use bioelectric activity recorded from muscles during contractions to classify actions. This technology has been used primarily for rehabilitation applications. In order to support the development of myoelectric interfaces for a broader range of human-machine interactions, we present an sEMG dataset obtained during key presses in a typing task. This fine-grained classification dataset consists of 16-channel bilateral sEMG recordings and key logs, collected from 19 individuals in two sessions on different days. We report baseline results on intra-session, inter-session and inter-subject evaluations. Our baseline results show that within-session accuracy is relatively high, even with simple learning models. However, the results on between-session and between-participant are much lower, showing that generalizing between sessions and individuals is an open challenge.

## Background & Summary

Effective interactions with complex devices requires high-throughput channels to convey user intent. Common computer peripherals such as keyboards, mice, and touchscreens are widespread and carefully designed for this purpose. Nonetheless, virtual, augmented, or mixed reality applications can call for more seamless and immersive interactions, in which reducing the need for manipulation of a physical peripheral may be desirable^1^. Moreover, people with disabilities might be limited in their ability to use these physical devices thus restricting their ability to interact. Neurotechnology offers an appealing avenue to solve this problem, thanks to its ability to provide direct access to a user’s intent by decoding their neural activity^2^.

Surface electromyography (EMG) recordings can be used as the basis for myoelectric control systems, which classify patterns of bioelectric activity to enable the user to select a particular gesture. This type of interface has been used extensively for the control of prosthetic limbs and other assistive technologies^3–5^. However, the gestures and myoelectric patterns required to accomplish a functional movement task (e.g. grasping an object) are different from those involved in the use of computer peripherals (e.g. typing).

A variety of EMG datasets for gesture classification are currently available. Widely used examples include the NinaPro database^6,7^, datasets based on mass-market EMG systems such as the Myo armband^8^, and high-density sEMG datasets such as CSL-HDEMG^9^ and CapgMyo^10^. These datasets have supported the development of numerous myoelectric classifiers, benefitting in recent years from the advent of deep learning methods^11^. In contrast, EMG datasets focused on movement tasks other than prosthetic control are scarce^12,13^. A greater variety of datasets would be beneficial to investigate the generalizability of myoelectric control approaches to new tasks. Furthermore, many recent classifiers report very high accuracies on standard datasets, such that ceiling effects may impede comparisons between methods; a more difficult classification task may provide opportunities for more differentiation and advancements.

We present a new EMG classification dataset consisting of recordings obtained during typing movements on a keyboard. Each key is a separate class, resulting in a 27-class problem with many similar classes and high relevance to the control of virtual or augmented reality systems with virtual keyboards. Data was collected on two separate days, providing the opportunity to examine inter-session generalization. We additionally report classification results with a simple baseline consisting of classical myoelectric classification techniques, using intra-subject, inter-subject and inter-session validation protocols.

The primary contribution of this work is providing the research community with a dataset containing a clear and diverse set of keypresses for each alphabetical key, with applications in virtual and mixed reality environments and more broadly in a variety of human-machine interaction scenarios. We also provide baselines on several experimental scenarios. These baselines show that a significant challenge in this domain is the inter-session and inter-user accuracy.

## Methods

19 uninjured individuals took part in the study (5 male, 14 female, 31 ± 7 years of age). The protocol was approved by the University Health Network Research Ethics Board (21-6137). Participants were recruited via study advertisements, and all provided written informed consent for the study procedures as well as for inclusion of the de-identified data in a public repository. The dataset includes only surface EMG recordings, which cannot be used on their own to re-identify any of the participants.

**Fig. 1:**
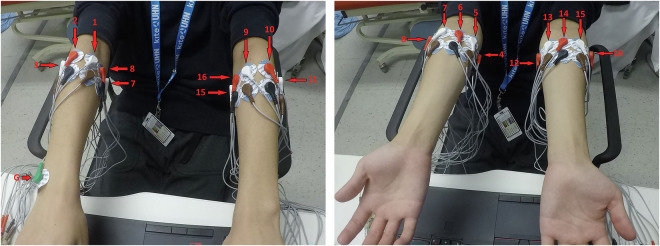
Electrode placements on pronated (left) and supinated (right) forearms.

Each participant repeated the entire protocol twice, on two separate days. Eight pairs of gel electrodes were placed on each arm, starting at the extensor carpi radialis and then rotating around the forearm (clockwise for the right arm, and counterclockwise for the left arm), for a total of 16 bipolar channels. A ground electrode was positioned on the styloid process at the wrist on the right arm. The placements are illustrated in Fig. 1. Surface EMG data was acquired at 2 kHz using a bipolar headstage and neural data acquisition board (C3313 and RHD2000, Intan Technologies, USA), and filtered between 10 Hz and 500 Hz.

Participants first conducted trials of 5 hand gestures (closed fist to open hand, extension of the wrist, forearm pronation and supination, pinching of the index fingers and thumbs, and both thumbs up) until clear signals were obtained, to verify the quality of the data acquisition. Participants were then asked to type each of 26 lowercase letters on a QWERTY keyboard, in randomized order. A diagram showing correct hand positioning and which finger to use for each letter was displayed to participants throughout the experiment. For each letter, participants were instructed to first tap the spacebar 5 times with their thumb. This motion provided a distinctive pattern in the recordings that could be later used to synchronize the EMG and keylogging data (see below). After the synchronization movement, participants pressed the target letter 10 times, following a metronome set to 75 beats per minute and returning to the base position between each press. This entire process was repeated twice for each letter, for a total of 20 repetitions.

During sEMG acquisition, a custom keylogging script was used to associate each key press with a timestamp. The keylogging data was synchronized with the sEMG data by aligning the 10 keypresses of the trial with the times of the keylogger. This was done by minimizing the difference between a spike train generated from the intervals of the keypresses and a spike train generated by a peak detection algorithm run on the sEMG signal after smoothing with a Gaussian filter. By minimizing the difference between the two spike trains, a lag between the two signals was found and used to synchronize the sEMG recording and the keylogger. This process was applied for each channel; the channel that gave the smallest difference to the keylog spike train after correction was taken as the lag. The minimum difference was recorded as the error for the selected lag. Once synchronized, the keylogger provided the specific timing and letter for each keystroke, thus creating labels for the sEMG data. The keylogger was also used to verify that the correct letters had been pressed. Some minor inconsistencies were observed in the keylogs, where not all letters were pressed 10 times by each participant. A complete list of the number of keypresses for each participant for each letter is provided with the dataset. In order to create a spacebar class, 20 spacebar presses were extracted at random across all recordings of a given participant from the synchronization spacebar presses used during the collection of the letter classes.

## Data Records

The dataset contains sEMG and keylogging data from a typing task with 19 uninjured participants across two sessions. The dataset has been deposited in the University of Toronto Dataverse repository^14^. The dataset contains a single zip folder. Once unzipped, there are 3 main folders and a series of supporting scripts. All data is stored in the CleanData folder (discussed below). Baseline results are included in the ClassificationResults folder. All processing scripts are stored in the Keypressemg folder. Other supporting scripts in the main directory include installation scripts for data processing (install.sh; install_using_poetry.sh, requirements.txt, prepare_data.sh), a data licence, and a ReadMe file.

All raw data is stored in the CleanData folder. The data for each participant is separated and labelled P1-P19. For each participant, trial folders T1 and T2 contain data from the two trials performed by the participant. Inside each trial folder, a Data folder contains the raw recordings for the sEMG signal across all the keypresses performed within a session. These are stored in .rhd format. Slicing and processing of these raw files is described in the ReadMe file. Additionally, a keylogs.txt file holds the raw key log data recorded along with the sEMG data. The keylog.txt file is a continuous stream of keylog entries across all typing tasks for that session. Finally, a LAG_TIMINGS.csv file contains the adjustments made to align the keylog and the EMG data for each recording. LAG_TIMINGS.csv has 3 columns, Filename, Lag and Error. The Filename column contains the file in the Data folder that the lag corresponds to. The Lag column is the difference in timing between the keylog and the start of the dataset. The Error column indicates the error value corresponding to the selected lag.

**Fig. 2:**
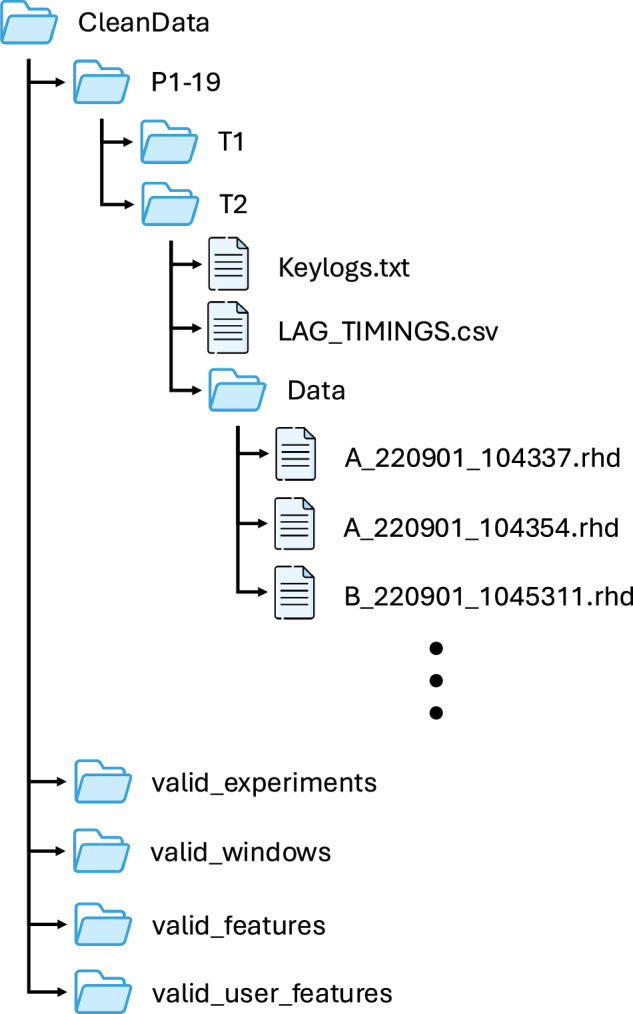
File structure for data storage.

The data structure is shown in Fig. 2. In addition to the raw data in CleanData there are 4 folders containing the data stored at different stages of pre-processing. These results are saved as .npy files for ease of access.The folder ‘CleanData/valid_experiments` provides all experiments that include the expected sequence of keypresses. The folder ‘CleanData/valid_windows` contains all the 0.2s windows extracted around the keypresses in each of these experiments. ‘CleanData/valid_features` contains the extracted time domain features from each window and ‘CleanData/valid_user_windows` contains these same features grouped by session. From the processed data, classification can be performed as described in the ReadMe included with the dataset.

## Technical Validation

### Data processing and classification

Once the files were aligned, preliminary testing for classification was conducted on the dataset. For classification, a SVM model with a radial basis function kernel was used across all tests. We note that we also experimented with deep neural networks trained on the raw signal and obtained better results with the hand-crafted features. This might be due to limited training data, or that network architectures need to be more carefully tailored to this data modality. The dataset contains the raw signals to support further research on deep neural networks on this dataset.

**Fig. 3:**
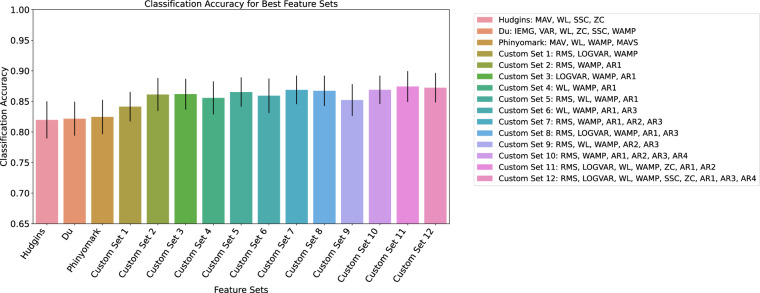
Mean classification accuracies per feature set with standard deviations.

First, different feature sets were tested for their classification accuracy. Common time domain EMG feature sets from studies by Phyniomark *et al*., Hudgins *et al*. and Du *et al*.^15–17^ were compared across all 19 participants. In addition, ten common EMG features (Root mean square (RMS), Logarithmic Varience (LOGVAR), Waveform Length (WL), Wilson Amplitude (WAMP), Standard Slope Change (SSC), Zero Crossings ZC, Autoregressive Coefficients 1-4 (AR1, AR2, AR3, AR4)) were identified from current literature and an exhaustive search of all feature combination was performed for a single participant. Twelve top feature sets from this sweep were then run across all 19 participants and the results were compared to the standard feature sets. For this comparison, the classification was performed using k-fold cross validation, with 4 folds each and 5 samples per letter. The result of the feature comparison is shown in Fig. 3. Feature comparison was performed with a 0.2s window length. The standard feature sets from the literature performed worse than the feature sets found through a feature sweep. The feature set with the best performance from this comparison was found to be (RMS, LOGVAR, WL, WAMP, ZC, AR1, AR2) with a classification accuracy of 87.4 ± 2.5 % averaged across the two test days for all 19 participants.

**Fig. 4:**
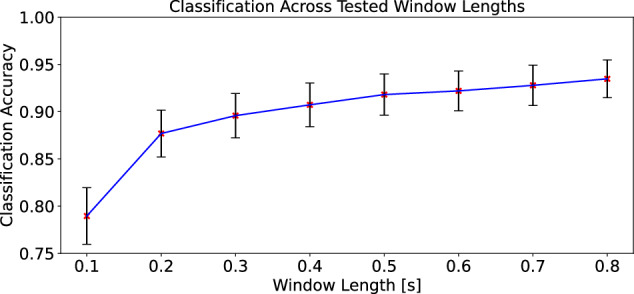
Mean classification accuracy across different window lengths with associated standard deviation.

The effect of window size for each keypress segment was also explored. Window sizes of 0.1 to 0.8 s in increments of 0.1 s were tested for classification accuracy and standard deviation. Similar to the feature analysis, k-fold cross validation was performed with 4 folds for this comparison. We note that an average typist generally types 40 - 60 words per minute which translates on average to 180+ keypresses per minute. Therefore, a timing window of less than 0.3 s should be used for real time application. A 0.2s window limits a typist to 300 characters per minute or roughly 60-80 words per minute. Figure 4 provides the results for different window lengths, which show that the classification accuracy increases with the window length size. However, we observe diminishing returns. We note that there is a trade-off between real-time usability of the algorithm and window length; a window length of 0.2 s is recommended for the baseline feature set to best balance the classification accuracy with the length of time needed per keypress.

Because the spacebar trials were extracted from the synchronization presses used when collecting the letter trials, precise movement instructions were not provided for the spacebar. The movements associated with this class are therefore expected to be more variable. Indeed, when we exclude the spacebar class, the accuracy on the 26-class problem using the 0.2 s window and feature set RMS, WAMP, AR1, AR2, AR3 increases to 90.2 ± 2.1 %.

**Fig. 5:**
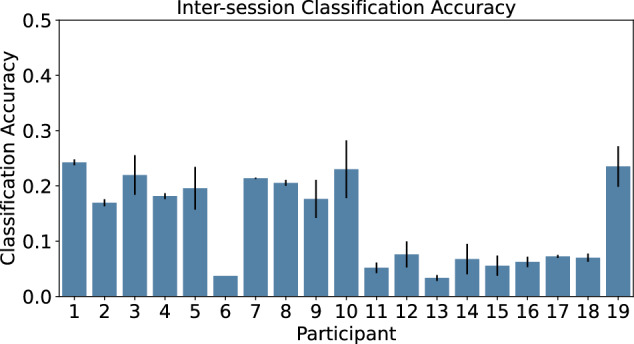
Classification Accuracy for training and testing on datasets from two separate days per participant.

Inter-session classification was also evaluated. For this analysis, we computed the classification accuracy when training on data from day one and testing on data from day two, and vice versa. The average of these two results was then taken to find an overall cross-test classification accuracy per participant. The feature set and window size used for this evaluation were the values obtained during the intra-session optimization. Inter-session comparisons per participant resulted in poorer performance than the intra-session evaluation (Fig. 5). The highest classification accuracy observed for a single participant was found for P1 of 24.26 ± 0.53%. The average classification accuracy across all participants for the inter-session comparison was 13.66 ± 1.71%.

**Fig. 6:**
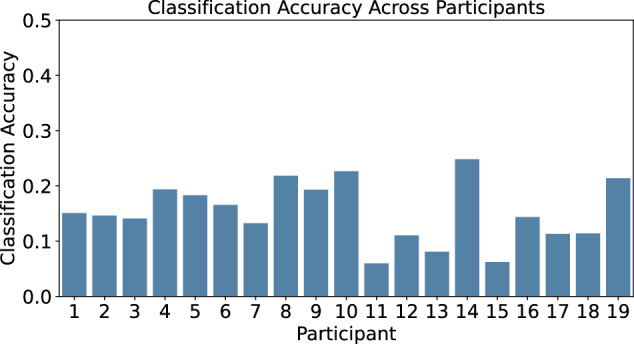
Classification Accuracy of a LOPO classification model.

A leave-one-participant-out approach (LOPO) was used to assess the transferability of classification models between participants. Once again, the feature set and window size for this evaluation were those obtained during the intra-session optimization. Similarly to the inter-session experiment, poor performance was observed for the LOPO models (Fig. 6). Here, the highest classification accuracy was found to be 24.81% for P14 with an average classification accuracy of 15.24 ± 5.08%. This poor result is not surprising as generalizing across sessions and individuals is a more challenging task. We also note the similarity between the inter-session accuracy and LOPO accuracy. As such, it seems that with current features different sessions are as unique as different patients.

### Federated Learning Experiments

We further explored how data can be shared across users via federated learning (FL). In federated learning, the users all train a joint model without aggregating their data in order to maintain privacy. They do however send gradients that can reveal some information about their dataset. We also experimented with personalized federated learning where each user has a unique personalized classifier instead of one joint classifier for all users.

As federated learning allows us to access more data, we tried to use a slightly more complex model, using a multilayer perceptron (MLP) over the extracted features. The FL experiments were conducted using the calculated features data (RMS, LOGVAR, WL, WAMP, ZC, AR1, AR2). Each 96-entry input vector corresponds to a single window, 6 calculated features for each of the 16 channels.

A few MLP architectures were tested. The results published here were achieved using an MLP that contains 4 Dense Blocks of sizes 96 → 192 → 192 → 192 → 48 followed by a Linear Layer 48 → 26. The first 3 Dense Blocks contain a Linear Layer followed by a ReLU activation and Dropout with probability 0.5. The 4th Dense Block uses an ELU activation and no Dropout.

Each FL round, 10 clients (out of 19) were sampled to receive the current global model. Each sampled client trained about 20 local epochs before sending back their gradients.

We experimented with one baseline federated learning algorithm, FedAvg^18^ and two personalized federated learning algorithms FedPer^19^ and pFedGP^20^. In FedAvg each round the server sends the model to several clients, they compute gradients on their data and the server aggregates all the gradients and updates the model. In FedPer all the network, besides the last layer, is shared and trained using FedAvg, while the last layer for each client is unique and is trained on its personal dataset. In pFedGP we use a Gaussian process with deep kernel learning, where the kernel is shared between clients and leaned with FedAvg while each clients predicts using a unique Gaussian process with its data on its dataset.

The results of the federated learning experiments are detailed in Table 1. We first notice that despite the higher capacity and access to more data, the intra-session accuracy is lower than the SVM trained on a single user. This again shows how transferring knowledge between users and sessions is a challenging problem. We hope that perhaps proper neural network design on this unique problem will address this issue. We also note that performance of the simple FedAvg model shows the best performance on the inter-session challenge, although the results are too low to be of use.

**Table 1:** Test performance averaged over 3 random seeds on key press federated learning classification tasks using calculated features from EMG signals.

	Inter-session (Acc.)	Intra-session (Acc.)
FedAvg	**18.90** ± **0.16**	53.30 ± 0.92
FedPer	14.39 ± 1.03	66.58 ± 1.01
pFedGP	12.45 ± 0.30	**74.49** ± **0.72**

### Implications of Validation Results for Open Challenges

The typing dataset presented here provides a valuable resource to improve myoelectric control strategies on a challenging problem with clear applications in human-machine interactions, going beyond common hand gesture recognition scenarios. We showed that classical machine learning methods performed relatively well on the intra-session problem for this task. A simple model on a standard feature set can achieve 87.4 ± 2.5% accuracy. However, there is still significant room for improvement with more complex models that must handle the limited amounts of data.

The relatively low classification accuracy observed when training and testing across the two sessions as well as on new participants provides interesting insight into the transferability of the learned classifier. Further work is warranted to explore improvements in both the inter-participant and inter-session classification scenarios. It would be particularly meaningful to improve the inter-session classification performance, which would remove the need for a lengthy calibration step each time the myoelectric interface is used. This is especially important if we wish to use the myoelectric interface for any commercial product. Inter-sessional variability of myoelectric classifiers is a well-known issue that is not specific to the present dataset. Palermo *et al* found that across 10 subjects, inter-sessional classification accuracies dropped by an average of 27.03% from training and testing with intra-session data to inter-session data^21^. There is evidence to show that if training data is collected over many sessions, the accuracy drop may be less^22^. Training with the limb in many different positions can also improve intersession accuracy^23^. Neither of these solutions are ideal, as increasing the number of training sessions reduces ease of implementation of the system, while multi positional training may be difficult for someone who is impaired. Recently, feature disentanglement methods have been used to find feature sets that are more robust to cross-session classification^24^. Most intriguingly, it has been demonstrated that large-scale training on thousands of individuals can also lead to generalizable classifiers^25^. Further work to optimize algorithms for cross-sessional EMG may provide sEMG control systems that are more viable in operation for fine-grained tasks.

This dataset provides a comprehensive and diverse set of sEMG recordings for fine-grained classification via the typing problem. There have been a few datasets published that are related to the presented work. One article contains keypress data from a single individual^12^; that work included 32 characters in a dataset obtained by transcribeing a recording of a conversation. Our dataset improves upon that work by containing data from 19 participants as opposed to one. Another dataset includes an sEMG typing dataset from 37 participants, with a slightly different focus putting emphasis on password security^13^. Other datasets include key presses but are more focused on detecting a typing task rather than identifying individual keypresses^26^.

Despite the increased complexity in our dataset, there are still some limitations, in particular the use of individual keypresses only rather than natural typing tasks. The well-controlled movements included in our dataset provide a valuable resource to characterize the expected performance achievable on this task. Nonetheless, the impact of variability in typing movements introduced during more natural typing scenarios will be an important avenue for future investigation. We also note the additional variety in movement for the spacebar class.

## Usage Notes

Instructions for installation of the necessary dependencies in a virtual environment, processing and classification are provided in the GitHub page for the dataset (see “Code availability”).

## Data Availability

The code for the baseline analyses is available at: https://github.com/ANSLab-UHN/sEMG-TypingDatabase
